# Improving the Quality of Ensiling High-Moisture Alfalfa with Peanut Vine in Different Additives: Fermentation, Nutritional Quality, and Microbial Communities

**DOI:** 10.3390/microorganisms13102228

**Published:** 2025-09-23

**Authors:** Haikuo Jia, Chunhui Wu, Zhenling Liu, Yu Sun, Ying He, Huan Chen, Xiaowei Zu, Lixin Wang, Yanxia Gao, Mingya Wang, Qiufeng Li

**Affiliations:** 1 College of Animal Science and Technology, Hebei Agricultural University, Baoding 071001, China; 18831481422@163.com (H.J.); wuchunhui2106@163.com (C.W.); yxgaohebau@126.com (Y.G.); 2 Hebei Provincial Animal Husbandry Station, Shijiazhuang 050036, China; 3 Animal Husbandry Station of the Department of Agriculture and Rural Affairs of Renze District in Xingtai City, Xingtai 054000, China

**Keywords:** by-product, cellulase, *Lactiplantibacillus plantarum*, mixed silage, tannin

## Abstract

Ensiling high-moisture alfalfa with peanut vine not only avoids alfalfa nutrient loss during the wilting stage but also maximizes the use of agricultural waste peanut vine. The appropriate mixed ratio of high-moisture alfalfa and peanut vine has been studied in our previous study. However, the effect of additives on improving the nutrition and fermentation quality of the mixed silage of alfalfa and peanut vine has not been investigated. This study aimed to assess the adaptation and association of *Lactiplantibacillus plantarum*, cellulase and tannin in the mixed silage of alfalfa and peanut vine alone or in combination on fermentation quality, chemical composition, and microbial communities. The harvested fresh alfalfa and dry peanut vine were cut into 2 cm lengths by a crop chopper and they were thoroughly mixed at a ratio of 7:3. The mixtures were treated with no addition (CK), *L. plantarum* (Lp, 1 × 10^6^ CFU/g fresh weight), cellulase (Ce, 5 g/kg fresh weight), tannin (Ta, 40 g/kg dry matter), and their combinations (LpCe, LpTa, CeTa, LpCeTa). After 45 days of fermentation, silage treated with Lp, Ce, and Ta had lower pH and ammonia-N (NH_3_-N) content and higher concentrations of lactic acid compared with the CK group. LpCeTa-treated silage inhibited protein degradation by reducing pH value and ammonia-N concentrations during ensiling processes. The LpCeTa group increased (*p* < 0.05) water-soluble carbohydrate (WSC) content and reduced (*p* < 0.05) acid detergent fiber and neutral detergent fiber contents in mixed silage. Furthermore, the LpCeTa group increased the relative abundance of *Lactobacillus* and decreased the relative abundance of *Enterococcus* and *Weissella* as compared with the CK group. Results of the current study indicated that the combined use of *L. plantarum*, cellulase, and tannin could serve as a promising strategy for the preservation of ensiling fresh alfalfa mixed with peanut vine and provide a reference for the re-utilization of by-product.

## 1. Introduction

Alfalfa (*Medicago sativa* L.) has been planted widely across the world due to its high content protein, many essential vitamins, and minerals [1,2]. Harvesting alfalfa as ensilage is a common conserving approach and can reduce the shortage of green feed for ruminants with restricted growth seasons [3]. However, its high natural moisture content makes alfalfa silage more difficult to produce successfully due to its higher susceptibility to spoilage by *Clostridia*, *Bacilli*, or *Enterobacter*, resulting in high dry matter (DM) loss, extensive proteolysis, and high butyric acid accumulation, which can reduce animal DM intake and increase milk contamination [4]. Therefore, mixing with other forage crops has become the conventional method used to improve the ensilability of alfalfa. This method could completely avoid unexpected rainfall events during wilting in actual production processes, reduce the nutrient losses caused by rain damage, increase the content of the silage fermentation substrate, and improve the success rate of the ensiling process, especially during rainy seasons [5].

Peanut vines, as a by-product of peanut cultivation, are characterized by high levels of crude protein (CP), digestible dry matter (DM), neutral detergent fiber (NDF), and acid detergent fiber (ADF) compared with other agricultural waste [6]. Our previous study indicated that alfalfa silage mixed with peat vine in a certain proportion avoided wilting in fields, increased the fermentation substrate and fermentation quality, and exhibited a positive associative effect to improve the utilization of feed [7]. This practice prevented peanut vine from being abandoned or burned and avoided environmental pollution and a waste of resources [7,8,9]. However, the silage quality of alfalfa mixed with peanut vine remains to be improved, especially its feed nutritional value.

Silage additives are used to improve silage fermentation characteristics and feed nutritional value [10]. Inoculating *L. plantarum* is one of the most useful methods of improving silage quality as it can further enhance lactic acid, thereby suppressing spoilage microorganisms [11]. Agarussi et al. [12] demonstrated that *L. plantarum* inoculation in alfalfa silage significantly enhanced lactic acid bacteria dominance while effectively suppressing the growth and reproduction of *Enterobacteria*. Inoculating *L. plantarum* also enhanced the fermentation quality through increased LA concentration and lowered pH in rice straw silage [13]. Cellulase, a complex enzyme that breaks down cellulose into monosaccharides, has been reported to increase lactic acid bacteria populations and improve silage fermentation quality [14,15]. Khota et al. [16] found that cellulase enzymes can promote fiber degradation and inhibit protein hydrolysis in tropical forage feed.

Tannic acid, a water-soluble polyphenolic compound, exhibits the capacity to bind proteins within silage and the rumen, thereby inhibiting proteolysis mediated by proteases [17]. However, the associated effects of the above-mentioned additives on silage have not been reported, and the most effective combination of additives remains unclear. This study hypothesizes that the addition of *L. plantarum*, cellulase, and tannin may alter the microbial community and enhance the fermentation properties of a mixture of ensiled high-moisture alfalfa with peanut vine.

The objective of this study was to investigate the effect of *L. plantarum*, cellulase, and tannin on fermentation characteristics, chemical compositions, and the microbial community and the correlations among them in high-moisture alfalfa silage mixed with peanut vine. This investigation aims to enhance the understanding of the functions of different additives in the ensiling process of high-moisture alfalfa mixed with peanut vine, thereby offering more comprehensive insights for optimizing the quality of mixed silage.

## 2. Materials and Methods

### 2.1. Forage Harvesting and Silage Preparation

Alfalfa (cultivar “WL358”) at the third-regrowth stage was manually harvested on 29 July 2022 from the experimental fields of Hebei Agricultural University, Baoding, Hebei, China (115°26′15″ E; 38°49′20″ N). Harvesting was using a sickle, leaving an 8 cm stubble height. Peanut vine was purchased from a village in Tang County (Baoding City, Baoding, China). Both alfalfa and peanut vine were sectioned into lengths of approximately 2 cm using a forage chopper. Then, alfalfa, without prior wilting, was mixed with peanut vine at ratios of 7:3. Eight treatment groups were prepared: (1) control (CK), sprayed with sterile distilled water; (2) *L. plantarum* at 10^6^ CFU/g FW (Lp); (3) cellulase at 5 g/kg FW (Ce); (4) tannin at 40 g/kg DM (Ta); (5) *L. plantarum* and cellulase (LpCe); (6) *L. plantarum* and tannin (LpTa); (7) cellulase and tannin (CeTa); and (8) *L. plantarum*, cellulase, and tannin (LpCeTa).

*L. plantarum* (10^10^ CFU/g) was purchased from Tianyi Biotechnology Co., Ltd., (Hangzhou, China); cellulase (10,000 U/g) was purchased from Xia Sheng Industrial Group (Yinchuan, China); and tannin (purity > 93%) was purchased from Henan Wanbang Chemical Technology Co., Ltd., (Shangqiu, China). Subsequently, approximately 400 g of mixed forage was thoroughly mixed and packed into nylon vacuum bags (25 × 35 cm; Zhejiang Mingke Plastic Industry Co., Ltd., Taizhou, China) and sealed with a vacuum extractor (Aoba brand P-290; Guangdong dongguan qingye company, Dongguan, China). In this study, there were 32 bags in total (8 treatments × 4 replicates = 32), they were kept at an ambient temperature (25~30 °C) and placed in a light-proof area. After 45 days of ensiling, the samples were used for analysis of fermentation performance, chemical composition, and changes in microbial diversity.

### 2.2. Fermentation Performance, Chemical Composition, and Microbial Analysis

The fermentation characteristics of silage were evaluated following a series of standardized procedures. Extraction fluid was prepared by immersing 20 g of fresh silage with 180 mL distilled water at 4 °C for 24 h. The filtrate was used to measure pH using a UB-7 precision acidity meter (Denver Instruments Co., Ltd. Denver, CO, USA) and its ammonia-N content was determined according to the method of Broderick et al. [18]. The volatile fatty acid (VFA) was determined by high-performance gas chromatography (Agilent 7890A gas chromatograph, Agilent, Santa Clara, CA, USA) employing a HP-INNOwax capillary-column chromatography (30.0 m × 320 μm × 0.5 μm). The carrier gas used for the samples was nitrogen.

Samples were oven-dried at 65 °C for 48 h to determine the dry matter (DM) content, and ground (10 mesh and 40 mesh screens) for chemical composition analysis. Crude protein (CP) was measured using the Kjeldahl method. Neutral detergent fiber (NDF) and acid detergent fiber (ADF) were determined by Van Soest et al. [19]. WSC was assessed using the anthrone–sulfuric acid method [20]. Relative feeding value (RFV) was calculated as described by Rohweder et al. [21]. The specific calculation method is defined as follows:

DDM = 88.9 − (0.779 × ADF) DMI = 120/NDF

RFV = (DDM × DMI)/1.29

where DDM is the digestible dry matter content in the dry matter. DMI is the dry matter intake.

Soluble protein (SP), NDF insoluble protein (NDIP), and acid detergent insoluble protein (ADIP) were analyzed as described by Licitra et al. [22]. Protein fractions (PA, PB1, PB2, PB3, and PC) were calculated according to the Cornell Net Carbohydrate and Protein System [23,24,25].

The silage bacterial community was analyzed by the next-generation sequencing technique, according to Wang et al. [26]. A universal primer (338F: 5′-ACTCCTACGGGAGGCAGCA-3′, 806R: 5′-GGACTACHVGGGTWTCTAAT-3′) was used to amplify the V3-V4 region of 16S rRNA gene from the genomic DNA. The purified polymerase chain reaction (PCR) products were sequenced using an Illumina platform (novaseq 6000, pe250, San Diego, CA, USA). Analysis of Microbiome Biological Information Using QIIME2 (Version 2024.5). Microbial communities were analyzed for α-diversity, β-diversity, and relative abundance via the personalbio online platform (https://www.genescloud.cn/home, accessed on 2 November 2022).

### 2.3. Statistical Analysis

The data from this experiment were recorded using Excel software (Microsoft Corporation, Redmond, WA, USA). One-way analysis of variance was performed in IBM SPSS Statistics version 20 (SPSS, Inc., Chicago, IL, USA) software. Graphs were generated using GraphPad Prism 9.5 (La Jolla, CA, USA) software. Duncan’s test was used to identify significant differences (*p* < 0.05) between treatments.

## 3. Results and Discussion

### 3.1. Chemical Compositions of Mixed Alfalfa with Peanut Vine Silage of Before Ensiling

The nutrient composition of mixed of alfalfa and peanut vine prior to ensiling are presented in Table 1. The dry matter (DM), crude protein (CP), neutral detergent fiber (NDF), acid detergent fiber (ADF) and water-soluble carbohydrate (WSC) in the alfalfa were 225.16 g/kg FW, 165.69 g/kg DM, 512.37 g/kg DM, 498.43 g/kg DM and 37.77 g/kg DM, respectively. The DM, NDF, and WSC of the peanut vine were higher than the alfalfa, but the CP and ADF were lower than the alfalfa. The DM content of the mixture of alfalfa and peanut vine was 436.2 g/kg FW. The CP and ADF contents of the mixture were 137.7 and 488.9 g/kg DM, respectively, which were lower than in a previous report by Bao et al. [27]. These discrepancies may be attributed to the influence of factors such as fertilization, harvesting season, planting density, and irrigation on forage nutritional composition [28]. The WSC content of the mixture was 38.2 g/kg DM, which was slightly higher than that of alfalfa ensiled alone. Overall, the results suggest that mixing peanut vine could potentially enhance the quality of alfalfa silage.

### 3.2. Effects of Different Additives on Fermentation Quality of Silage Mixed Alfalfa with Peanut Vine

The fermentation profile of mixed silage is shown in Table 2. The pH value, NH_3_-N, lactic acid, acetic acid, and butyric acid content were significantly (*p* < 0.05) affected by different additives. The pH value, NH_3_-N, and VFA are generally used as critical indicators of assessing silage fermentation quality [29]. The pH value of the Lp, Ce, and Ta groups were significantly decreased compared with the CK group. Moreover, the NH_3_-N content was significantly lower in the Lp and Ce groups, while the lactic acid content was significantly higher in these groups. However, the combination of *L. plantarum*, cellulase, or tannin further improved fermentation quality compared with the silage treated with additives alone. Particularly, the pH values of the LpCe and LpCeTa groups were significantly lower than those of the LP, Ta, LpTa, and CeTa groups (*p* < 0.05). At the same time, the lactic acid content in the LpCe and LpCeTa groups was significantly higher than that in these groups. *L. plantarum* and cellulase collectively enhance the fermentation process by increasing lactic acid production and reducing pH, while tannin inhibits the growth of undesirable microorganisms and reduces protein degradation. David et al. [30] reported that the combined addition of *L. plantarum* and cellulase was helpful for maintaining fibrous substances in mulberry leaves and reducing CP loss and NH_3_-N production in silage. In addition, the NH_3_-N content in the LpCe group was significantly lower than that in the Ce, Ta, LpTa, CeTa, and LpCeTa groups (*p* < 0.05). This reduction suggests that the combined of *L. plantarum* and cellulase hindered the activity of proteolytic enzymes, consequently reducing the rate of protein breakdown.

### 3.3. Effects of Additives on Chemical Composition of Mixed Alfalfa and Peanut Vine Silage

The chemical composition of mixed silage is shown in Table 3. Different additives had significant effects on chemical composition (*p* < 0.05). The CP contents in the Lp and Ce groups were significantly higher than that in the CK group (*p* < 0.05), while NDF and ADF contents decreased in the Lp and Ce groups; this phenomenon may be due to the addition of cellulase, which breaks down the indigestible polymers such as cellulose and hemicellulose into soluble sugars, thereby promoting the growth of beneficial microorganisms. WSC plays a crucial role in shaping fermentation characteristics and microbial activities in silage [31]. The WSC contents in the Ce and Ta groups were higher than that in the CK group (*p* < 0.05). Research conducted by Ji et al. [32] demonstrated that silage supplemented with cellulase retained a higher concentration of WSC, thereby facilitating rapid fermentation by lactic acid bacteria (LAB), which subsequently became the dominant microbial population. This dominance effectively inhibited the utilization of WSC by undesirable bacteria, which was consistent with the results of this experiment. The RFV in the CK group was significantly (*p* < 0.05) lower than the Lp and Ce groups. In this study, the LpCeTa group resulted in the highest crude protein content and RFV, as well as the lowest ADF content compared with the individual or other combined additive groups. This phenomenon may be attributed to the rapid acidification induced by *L. plantarum*, which lowers the pH and suppresses protein degradation by deleterious microorganisms.

### 3.4. Effects of Additives on Nitrogen Fractions and CNCPS Composition of Mixed Alfalfa and Peanut Vine Silage

The nitrogen fractions of mixed silage are shown in Table 4. The nitrogen fractions were significantly different with additives (*p* < 0.05). Notably, the TP of the Ce and Ta groups were significantly higher than that in the CK and Lp groups (*p* < 0.05), while the NPN contents were significantly lower than that in the CK and Lp groups. Research conducted by Chen et al. [33] demonstrated that the addition of tannin decreased the proportion of PA and PB1, while increasing the proportion of PB and PB2 compared with the control. Tabacco et al. [34] also reported that the tannin addition reduced NPN, ammonia, and Met values in alfalfa silage. Moreover, the SP content in the Ce group was significantly higher than that in the LpCe, Ta, LpTa, CeTa, and LpCeTa groups (*p* < 0.05). The ADIP content of the Ta was significantly higher than that in the CK and other groups (*p* < 0.05). The addition of *L. plantarum* and tannin effectively suppressed the proliferation of detrimental microorganisms like *Clostridium* and *Enterobacteriaceae*, thereby reducing protein degradation. The NDIP content in the LpTa group was significantly higher than in the CK and other groups (*p* < 0.05).

The CNCPS composition of mixed silage is provided in Table 5. The PA content in the CK and Lp groups was significantly higher compared with the other groups (*p* < 0.05). Furthermore, the PB1 levels in the Ce group were significantly higher than that in the CK and other groups. The PB2 levels in the LpCeTa group were significantly higher than those in the CK, Lp, Ce, and LpCe groups (*p* < 0.05). In contrast, no significant differences in PB2 levels were observed among the Ta, LpTa, and CeTa groups. The addition of tannin resulted in a significant decrease in NPN content and a notable increase in NDIP, ADIP, and PC content, this phenomenon is likely attributable to the addition of tannin combining with protein to form a tannin–protein complex, effectively inhibiting protein degradation, reducing NPN production, and increasing the PC fraction. Min et al. [35] found that the addition of tannic acid could increase the rumen protein content of ruminants.

### 3.5. Bacterial Community of Mixed Alfalfa and Peanut Vine Silage

**Figure 1:**
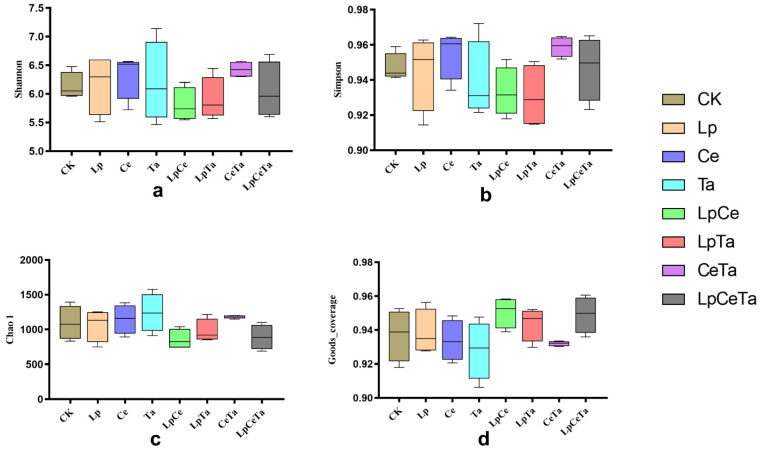
Changes in Alpha-diversity of the bacterial community. (**a**) Shannon, (**b**) Simpson, (**c**) Chao1, (**d**) Goods_coverage.

The consistent pattern of changes in alpha diversities of mixed silages with different additives (Figure 1). The average Goods_coverage value for all samples was over 0.90.

**Figure 2:**
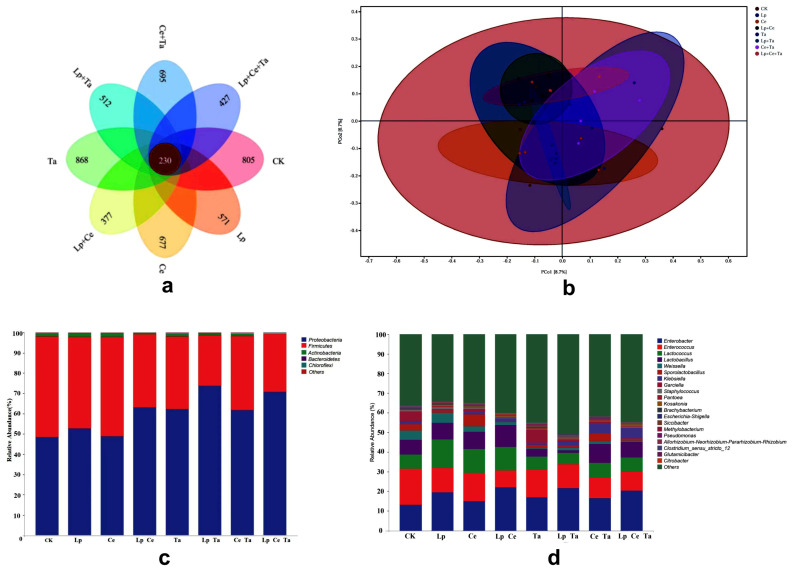
Changes in bacterial communities. (**a**) Venn diagram, (**b**) beta diversity, (**c**) relative abundance of bacterial phylum, and (**d**) relative abundance of bacterial genus.

The common and unique operational taxonomic units (OTUs) between different treatments of mixed silage as influenced by additives are shown in Figure 2a. The Venn diagram illustrates that 230 OTUs were shared among all treatment groups, whereas the majority of OTUs were specific to individual treatments. The PCoA plot for sequence similarities using the unweighted UniFrac displayed a clear clustering of the bacterial community by different treatments (Figure 2b). The microbial relative abundances of mixed alfalfa and peanut vine silage with different additives are shown in Figure 2c,d. Generally, *Proteobacteria* and *Firmicutes* were the main families across all treatments at the phylum level (Figure 2c), which is consistent with the findings reported by Ogunade et al. [36]. Tannin exhibited higher resistance against Gram-positive bacteria. In this study, the relative abundance of *Firmicutes* decreased in the LpCeTa group, a decrease that may be attributed to the formation of tannin–protein complexes, which effectively inhibit protein degradation, reduce the production of non-protein nitrogen (NPN), and increase the cell wall-associated protein fraction. Moreover, the relative abundance of *Actinobacteria* was reduced in the LpCeTa group compared with the other treatments. This phenomenon can be attributed to the production of lactic acid by lactic acid bacteria, which decreases the pH and creates an acidic environment that inhibits the growth of anaerobic microorganisms. Additionally, tannins possess antibacterial properties, effectively suppressing the proliferation of various microorganisms. Figure 2d shows the most abundant genera at the taxonomic level of the genus across different treatment groups. Previous research has indicated that *Enterobacter*, *Enterococcus*, *Lactococcus*, and *Lactobacillus* collectively constitute the primary flora in alfalfa silage at the genus level [37]. This dominance can be primarily attributed to the low pH and anaerobic conditions characteristic of the silage environment, which are conducive to the proliferation of these bacterial groups [38]. The increase in the abundance of *Lactobacillus* can rapidly produce a large amount of lactic acid, leading to a rapid drop in pH value and inhibiting the activity of putrefactive bacteria and some Clostridium, thereby reducing the deamination of proteins and hydrolytic loss, and improving the retention rate of CP. In comparison with the CK treatment, the LpCeTa group increased the relative abundance of *Lactobacillus* and decreased the relative abundances of *Enterococcus* and *Weissella*. This indicates that the addition of cellulase may alleviate the suppressive impact of tannins on *Lactobacillus*, thereby improving the fermentation process. Cellulase facilitates the breakdown of certain polysaccharides into WSC, providing essential nutrients for lactic acid bacteria and resulting in a rapid proliferation of bacterial populations.

### 3.6. Correlation Analysis Between Bacterial Community and Various Parameters

**Figure 3:**
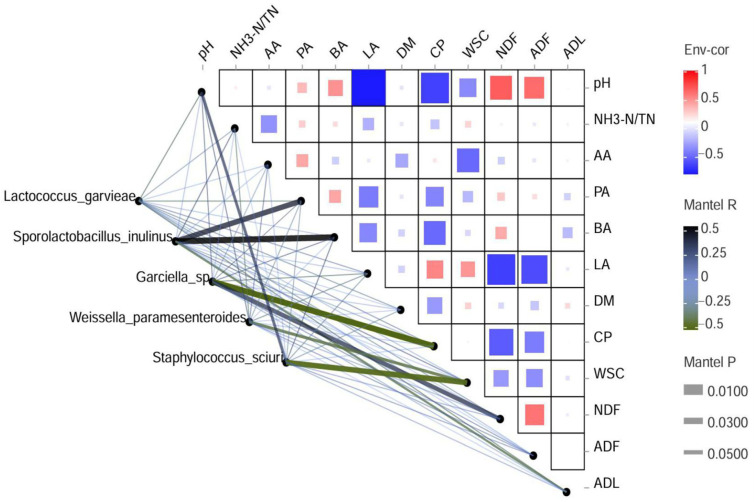
Correlation of bacteria community composition and various indicators in silage. The triangles in the upper-right corner represent the correlation between different indicators, and the color of each square indicates the positive and negative correlation coefficients between these various parameters. The lines in the lower-left corner represent the correlation of bacterial community composition with each indicator obtained through partial Mantel tests. AA, Acetic acid; PA, Propionic acid; BA, Butyric acid; LA, Lactic acid; DM, dry matter; CP, crude protein; WSC, water-soluble carbohydrate; NDF, neutral detergent fiber; ADF, acid detergent fiber; ADL, Acid detergent lignin.

A Spearman correlation between pH, NH_3_-N, VFA, DM, CP, WSC, NDF, ADF, and ADL contents and kinetics of the top five species during the ensiling was generated and is illustrated in Figure 3. The analysis revealed a positive correlation between *Lactococcus garvieae* and LA and CP, while a negative correlation was observed with silage pH, PA, and ADL. This phenomenon can be attributed to the production of lactic acid from the fermentation of WSC, leading to a decrease in pH, which inhibits the growth of detrimental microorganisms and consequently promotes the proliferation of *Lactococcus garvieae*, which is consistent with the findings reported by Chen et al. [39]. Furthermore, *Sporolactobacillus inulinus* exhibited a significant correlation with PA and BA. The content of WSC is highly correlated with *Weissella_paramesenteroides*. *Weissella paramesenteroides* efficiently utilizes WSC, playing a crucial role in silage acidification and stabilization. Chen et al. [40] have demonstrated that the presence of specific sugars like fructose and galactose can increase the *Weissella_paramesenteroides* population in forage crops, potentially influencing the silage fermentation quality, which was consistent with the results of the present study.

## 4. Conclusions

This study revealed that the combination addition of *Lactiplantibacillus plantarum*, cellulase, and tannin is more effective than addition alone and exhibits associated effects in high-moisture alfalfa silage mixed with peanut vine. During the silage process, the combination addition decreased pH value, butyric acid, NH_3_-N concentrations, NDF, ADF, non-protein nitrogen, and PA while increasing lactic acid, CP contents, WSC, and TP in alfalfa and peanut vines silage by increasing beneficial microorganisms like *Lactobacillus* and decreasing the relative abundance of undesirable bacteria such as *Enterococcus* and *Weissella*. This study provides important information for high-moisture alfalfa silage and for high value utilization of peanut vines.

## Figures and Tables

**Table 1:** Chemical compositions of fresh alfalfa and peanut vine before ensiling.

Items	Alfalfa	Peanut Vine	Mixture
Dry matter (g/kg FW)	225.2 ± 0.76	928.6 ± 0.75	436.2 ± 0.48
Crude protein (g/kg DM)	165.7 ± 0.34	72.4 ± 0.77	137.7 ± 0.07
Neutral detergent fiber (g/kg DM)	512.4 ± 4.24	575.2 ± 7.69	531.2 ± 1.87
Acid detergent fiber (g/kg DM)	498.4 ± 2.42	466.7 ± 2.73	488.9 ± 1.48
Water-soluble carbohydrate (g/kg DM)	37.8 ± 0.55	39.3 ± 1.09	38.2 ± 0.20
Crude ash (g/kg DM)	127.3 ± 0.47	86.5 ± 0.87	115.01 ± 0.20

Note: FW: fresh weight; DM: dry matter.

**Table 2:** Effects of different additives on fermentation indexes of mixed alfalfa and peanut vine silage.

	pH	Lactic Acid (g/kg DM)	NH3-N (g/kg TN)	Acetic Acid (g/kg DM)	Propionic Acid (g/kg DM)	Butyric Acid (g/kg DM)
CK	5.87 ± 0.29 a	9.40 ± 0.33 c	44.49 ± 0.19 a	17.15 ± 0.51 abc	1.78 ± 0.19	0.70 ± 0.03 a
Lp	5.18 ± 0.16 bc	21.90 ± 0.16 b	34.81 ± 0.29 de	20.43 ± 0.31 a	1.28 ± 0.08	0.31 ± 0.01 bc
Ce	4.96 ± 0.19 cd	42.78 ± 0.40 a	39.02 ± 0.38 bcd	14.99 ± 0.27 bc	0.79 ± 0.02	0.48 ± 0.03 ab
Ta	5.34 ± 0.09 bc	22.50 ± 0.44 b	42.87 ± 0.40 ab	13.53 ± 0.14 c	1.20 ± 0.03	0.26 ± 0.01 bc
LpCe	4.74 ± 0.06 d	45.77 ± 0.17 a	31.36 ± 0.11 e	17.42 ± 0.13 abc	0.58 ± 0.01	0.13 ± 0.00 c
LpTa	5.40 ± 0.07 b	20.67 ± 0.06 b	38.65 ± 0.18 bcd	17.44 ± 0.24 abc	1.71 ± 0.05	0.31 ± 0.01 bc
CeTa	5.06 ± 0.09 cd	26.02 ± 0.67 b	40.59 ± 0.23 abc	15.82 ± 0.21 bc	1.52 ± 0.08	0.44 ± 0.01 b
LpCeTa	4.79 ± 0.03 d	45.65 ± 0.41 a	37.47 ± 0.25 cd	19.51 ± 0.20 ab	1.27 ± 0.02	0.30 ± 0.02 bc

Note: CK: no additives; Lp: *L. plantarum*; Ce: cellulase; Ta: tannin; LpCe: *L. plantarum* and cellulase; LpTa: *L. plantarum* and tannin; CeTa: cellulase and tannin; LpCeTa: *L. plantarum*, cellulase and tannin; TN: total nitrogen; NH_3_-N: ammonia-N. ^a–e^: Different lowercase letters in the column data indicate a significant difference (*p* < 0.05), while the same letter or no letter indicates no significant difference (*p* > 0.05).

**Table 3:** Effects of additives on chemical composition of mixed alfalfa and peanut vine silage.

	DM (g/kg FW)	CP (g/kg DM)	NDF (g/kg DM)	ADF (g/kg DM)	EE (g/kg DM)	WSC (g/kg DM)	RFV
CK	420.6 ± 0.22 bc	131.5 ± 0.31 d	575.7 ± 3.33 a	476.1 ± 2.29 a	20.0 ± 0.03 c	19.2 ± 0.11 d	840.2 ± 7.21 d
Lp	417.2 ± 0.75 c	140.4 ± 0.17 ab	538.4 ± 2.09 b	451.2 ± 1.43 bc	27.4 ± 0.06 a	16.4 ± 0.07 e	929.2 ± 1.88 cd
Ce	426.4 ± 0.38 ab	139.4 ± 0.17 bc	529.7 ± 1.67 b	427.6 ± 1.38 d	27.1 ± 0.12 a	30.1 ± 0.13 a	977.3 ± 4.35 ab
Ta	429.4 ± 0.19 a	129.6 ± 0.06 d	554.7 ± 2.64 ab	463.5 ± 0.45 ab	22.4 ± 0.10 b	26.6 ± 0.03 b	886.6 ± 3.85 cd
LpCe	414.8 ± 0.48 c	141.6 ± 0.17 ab	531.7 ± 2.27 b	445.5 ± 1.33 bcd	27.4 ± 0.06 a	26.9 ± 0.14 b	949.1 ± 2.53 ab
LpTa	430.7 ± 0.06 a	137.3 ± 0.13 c	535.6 ± 1.85 b	437.3 ± 0.59 cd	21.9 ± 0.24 bc	24.4 ± 0.09 c	953.1 ± 2.53 ab
CeTa	425.6 ± 0.37 ab	140.1 ± 0.10 ab	544.5 ± 1.09 ab	439.1 ± 1.15 cd	25.7 ± 0.25 a	27.1 ± 0.11 b	934.7 ± 2.70 abc
LpCeTa	417.5 ± 0.25 c	142.5 ± 0.04 a	521.5 ± 1.11 b	425.9 ± 0.34 d	22.7 ± 0.13 b	23.2 ± 0.17 c	994.2 ± 1.84 a

Note: CK: no additives; Lp: *L. plantarum*; Ce: cellulase; Ta: tannin; LpCe: *L. plantarum* and cellulase; LpTa: *L. plantarum* and tannin; CeTa: cellulase and tannin; LpCeTa: *L. plantarum*, cellulase and tannin. FW: fresh matter; DM: dry matter; CP: crude protein; NDF: neutral detergent fiber; ADF: acid detergent fiber; EE: ether extract; WSC: water-soluble carbohydrate; RFV: relative feeding value. ^a–e^: Different lowercase letters in the column data indicate a significant difference (*p* < 0.05), while the same letter or no letter indicates no significant difference (*p* > 0.05).

**Table 4:** Effects of additives on nitrogen fractions of mixed alfalfa and peanut vine silage.

	TP (g/kg CP)	NPN (g/kg CP)	SP (g/kg CP)	ADIP (g/kg CP)	NDIP (g/kg CP)
CK	437.4 ± 1.23 d	562.6 ± 1.23 a	578.7 ± 1.14 ab	141.8 ± 0.25 d	162.6 ± 0.37 c
Lp	446.7 ± 1.12 d	553.4 ± 1.12 a	575.6 ± 1.19 ab	132.3 ± 0.32 e	144.6 ± 0.36 e
Ce	471.7 ± 0.86 c	528.3 ± 0.86 b	582.9 ± 0.93 a	136.7 ± 0.14 e	140.4 ± 0.19 e
Ta	478.7 ± 1.06 bc	521.3 ± 1.06 bc	538.0 ± 0.93 c	168.1 ± 0.28 a	171.8 ± 0.29 b
LpCe	471.0 ± 0.77 c	530.0 ± 0.77 b	565.8 ± 0.66 b	145.4 ± 0.55 cd	168.8 ± 0.72 b
LpTa	495.8 ± 0.79 a	504.2 ± 0.79 d	525.5 ± 0.80 c	161.7 ± 0.25 b	177.7 ± 0.43 a
CeTa	495.7 ± 1.26 a	504.4 ± 1.26 d	534.3 ± 1.53 c	146.5 ± 0.36 cd	159.7 ± 0.15 c
LpCeTa	490.0 ± 0.99 ab	510.1 ± 0.99 cd	538.3 ± 1.07 c	146.9 ± 0.22 c	152.1 ± 0.09 d

Note: CK: no additives; Lp: *L. plantarum*; Ce: cellulase; Ta: tannin; LpCe: *L. plantarum* and cellulase; LpTa: *L. plantarum* and tannin; CeTa: cellulase and tannin; LpCeTa: *L. plantarum*, cellulase and tannin. TP, True protein; NPN, non-protein nitrogen; SP, soluble protein; ADIP, acidic detergent insoluble protein; NDIP, neutral detergent insoluble protein. ^a–e^: Different lowercase letters in the column data indicate a significant difference (*p* < 0.05), while the same letter or no letter indicates no significant difference (*p* > 0.05).

**Table 5:** Effects of additives on CNCPS components of mixed alfalfa and peanut vine silage.

	PA (g/kg CP)	PB1 (g/kg CP)	PB2 (g/kg CP)	PB3 (g/kg CP)	PC (g/kg CP)
CK	562.6 ± 1.23 a	16.1 ± 0.09 e	257.6 ± 1.39 d	20.8 ± 0.13 a	141.8 ± 0.25 d
Lp	553.4 ± 1.12 a	22.2 ± 0.08 d	271.9 ± 2.01 cd	12.3 ± 0.27 c	132.3 ± 0.32 e
Ce	528.3 ± 0.86 b	54.6 ± 0.09 a	281.3 ± 0.93 bc	3.7 ± 0.11 d	136.7 ± 0.14 e
Ta	521.3 ± 1.06 bc	16.7 ± 0.17 e	288.6 ± 1.16 abc	3.6 ± 0.11 d	168.1 ± 0.28 a
LpCe	529.0 ± 0.77 b	36.8 ± 0.16 b	270.0 ± 1.32 cd	23.5 ± 0.28 a	145.4 ± 0.55 cd
LpTa	504.2 ± 0.79 d	21.3 ± 0.20 d	295.5 ± 0.76 ab	16.0 ± 0.26 b	161.7 ± 0.25 b
CeTa	504.4 ± 1.26 d	29.9 ± 0.35 c	305.8 ± 1.11 a	13.3 ± 0.23 bc	146.5 ± 0.36 cd
LpCeTa	510.1 ± 0.99 cd	28.2 ± 0.17 c	300.4 ± 1.61 ab	5.2 ± 0.16 d	146.9 ± 0.22 c

Note: CK: no additives; Lp: *L. plantarum*; Ce: cellulase; Ta: tannin; LpCe: *L. plantarum* and cellulase; LpTa: *L. plantarum* and tannin; CeTa: cellulase and tannin; LpCeTa: *L. plantarum*, cellulase and tannin. PA, the proportion of NPN to CP; PB1, the proportion of rapidly degrading protein to CP; PB2, the proportion of moderately degraded protein to CP; PB3, the proportion of slow-degrading protein to CP; PC, the proportion of bound protein to CP. ^a–e^: Different lowercase letters in the column data indicate a significant difference (*p* < 0.05), while the same letter or no letter indicates no significant difference (*p* > 0.05).

## Data Availability

The original contributions presented in this study are included in the article. Further inquiries can be directed to the corresponding authors.

## Abbreviations

CK: no additives; Lp: *Lactiplantibacillus plantarum*; Ce: cellulase; Ta: tannin; FW: fresh weight; DM: dry matter; TN: total nitrogen; NH_3_-N: ammonia-N; CP: crude protein; NDF: neutral detergent fiber; ADF: acid detergent fiber; EE: ether extract; WSC: water-soluble carbohydrate; RFV: relative feeding value; TP, True protein; NPN, non-protein nitrogen; SP, soluble protein; ADIP, acidic detergent insoluble protein; NDIP, neutral detergent insoluble protein; PA, the proportion of NPN to CP; PB1, the proportion of rapidly degrading protein to CP; PB2, the proportion of moderately degraded protein to CP; PB3, the proportion of slow-degrading protein to CP; PC, the proportion of bound protein to CP.
